# Cannonball Metastases of the Lung: An Unusual Initial Manifestation of Endometrial Carcinoma

**DOI:** 10.1002/rcr2.70455

**Published:** 2026-01-05

**Authors:** Midila Bapineni, Shivendra Tangutoori, Naga Vamsi Krishna Machineni, Kamlesh Sajnani, Maneesh Gaddam

**Affiliations:** ^1^ Department of Internal Medicine Appalachian Regional Healthcare Harlan Kentucky USA; ^2^ Department of Hematology and Oncology Appalachian Regional Healthcare Harlan Kentucky USA; ^3^ Department of Pulmonary, Critical Care and Sleep Medicine Appalachian Regional Healthcare Hazard Kentucky USA

**Keywords:** cannonball lesions, endometrial carcinoma, lung nodules, malignancy, metastasis

## Abstract

Endometrial carcinoma is the most common gynecologic malignancy in developed countries and is typically diagnosed at an early stage, with distant metastases uncommon at presentation. Pulmonary involvement occurs in less than 5% of cases and usually manifests as small nodules or interstitial disease; the appearance of multiple, large, round ‘cannonball’ metastases is exceptionally rare. We report the case of a 60‐year‐old woman who presented with cough and dyspnea. Imaging revealed numerous bilateral pulmonary ‘cannonball’ nodules, mediastinal lymphadenopathy, hepatic lesions, and an enlarged uterus. Endometrial biopsy confirmed endometrioid adenocarcinoma, staged as FIGO IV‐B. The patient underwent chemotherapy with carboplatin and paclitaxel, resulting in significant regression of pulmonary and hepatic metastases and symptomatic improvement, followed by maintenance therapy with anastrozole that achieved stable disease on surveillance. This case highlights the importance of recognising cannonball pulmonary metastases as a potential, albeit rare, initial presentation of endometrial carcinoma, thereby guiding timely diagnosis and treatment.

## Graphical abstract

Cannonball pulmonary metastases, typically linked to renal cell carcinoma or choriocarcinoma, are rare in gynecologic cancers. Endometrial carcinoma seldom spreads to the lungs and usually presents as small nodules, making recognition of this atypical pattern crucial to avoid diagnostic delay.

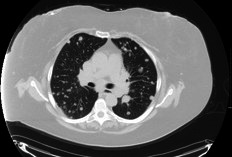

## 1 Introduction

Endometrial carcinoma is the most common gynecologic malignancy in developed countries and a leading cancer among women worldwide [1]. Most cases are detected early due to abnormal uterine bleeding, especially in postmenopausal women, and have favorable outcomes with five‐year survival exceeding 90%. In contrast, advanced or metastatic disease is less common and carries a markedly worse prognosis, with pulmonary metastases reported in only 2.3%–4.7% of patients [2, 3]. These metastases usually appear as small nodules or interstitial changes; large, well‐defined ‘cannonball’ lesions are exceedingly rare. The term ‘cannonball metastases’ describes multiple, round, sharply circumscribed pulmonary nodules resembling historical artillery fire and is classically associated with renal cell carcinoma, choriocarcinoma, and occasionally breast or colorectal carcinoma [4, 5]. Infectious or autoimmune conditions may mimic this radiographic pattern, but gynecologic primaries seldom do. We report a rare case of endometrial carcinoma that initially presented with respiratory symptoms, where progressive cough and dyspnea led to the discovery of widespread cannonball pulmonary metastases on imaging.

## 2 Case Report

A 60‐year‐old woman with no prior medical history and a lifetime non‐smoker presented to her primary physician with a persistent nonproductive cough. She was empirically treated with antibiotics for presumed bronchitis, but her symptoms persisted and gradually progressed to exertional dyspnea. She denied fever, chills, hemoptysis, chest pain, weight loss, or systemic complaints.

**FIGURE 1:**
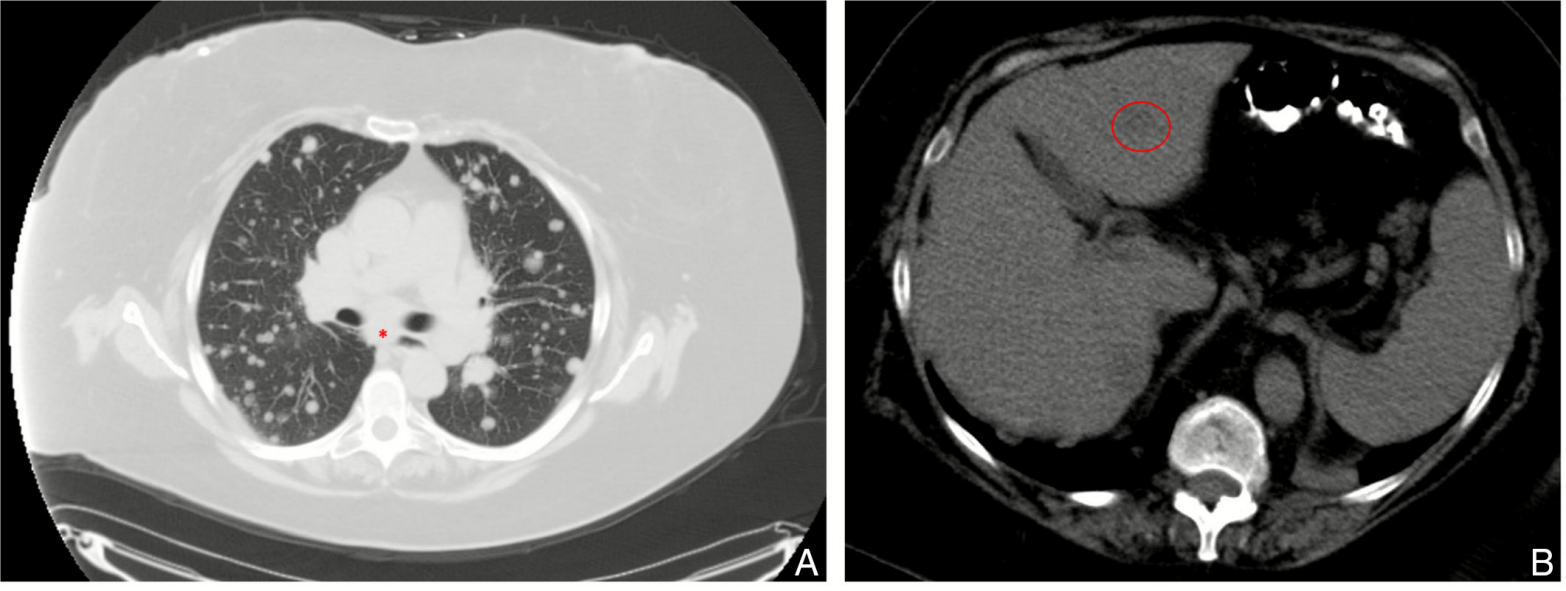
(A) Multiple bilateral “cannonball” pulmonary nodules with enlarged subcarinal lymphadenopathy(*). (B) Low‐attenuation lesion in the left hepatic lobe.

Chest radiograph was abnormal, prompting a computed tomography (CT) scan that demonstrated numerous, well‐circumscribed pulmonary nodules ranging from a few millimetres to 2 cm, distributed both peripherally and diffusely, along with mediastinal lymphadenopathy measuring up to 2.8 cm in the subcarinal region (Figure 1A). CT of the abdomen showed low‐attenuation hepatic lesions measuring 1.3 and 1.9 cm, as well as an enlarged uterus (6.4 cm) (Figure 1B), raising suspicion for metastatic disease.

At this stage, the differential diagnosis for multiple pulmonary nodules included metastatic malignancy, infections, inflammatory disorders, and vasculitides such as granulomatosis with polyangiitis. However, the clinical context strongly supported a metastatic aetiology. The patient lacked infectious symptoms, had no risk factors or features suggestive of tuberculosis or GPA, and the ancillary imaging findings were highly consistent with metastatic disease. Given these considerations, her respiratory symptoms were attributed to metastatic involvement, and additional laboratory evaluation was not pursued at that time.

Transvaginal ultrasound confirmed an enlarged uterus with thickened endometrium (0.93 cm). Endometrial biopsy revealed endometrioid adenocarcinoma with squamous differentiation. Molecular profiling demonstrated oestrogen receptor (90%) and progesterone receptor (75%) positivity, MMR proficiency, MSI stability, HER2 negativity, PTEN exon 5 mutation, PIK3R1 positivity, and POLE negativity.

At oncology consultation, her Eastern Cooperative Oncology Group (ECOG) performance status was 2 with a modified Medical Research Council (MMRC) grade 3 dyspnea. Apart from mild post‐biopsy vaginal bleeding, she denied pelvic pain or systemic symptoms. She was staged as cT1N0M1, corresponding to FIGO stage IV‐B endometrial carcinoma.

**FIGURE 2:**
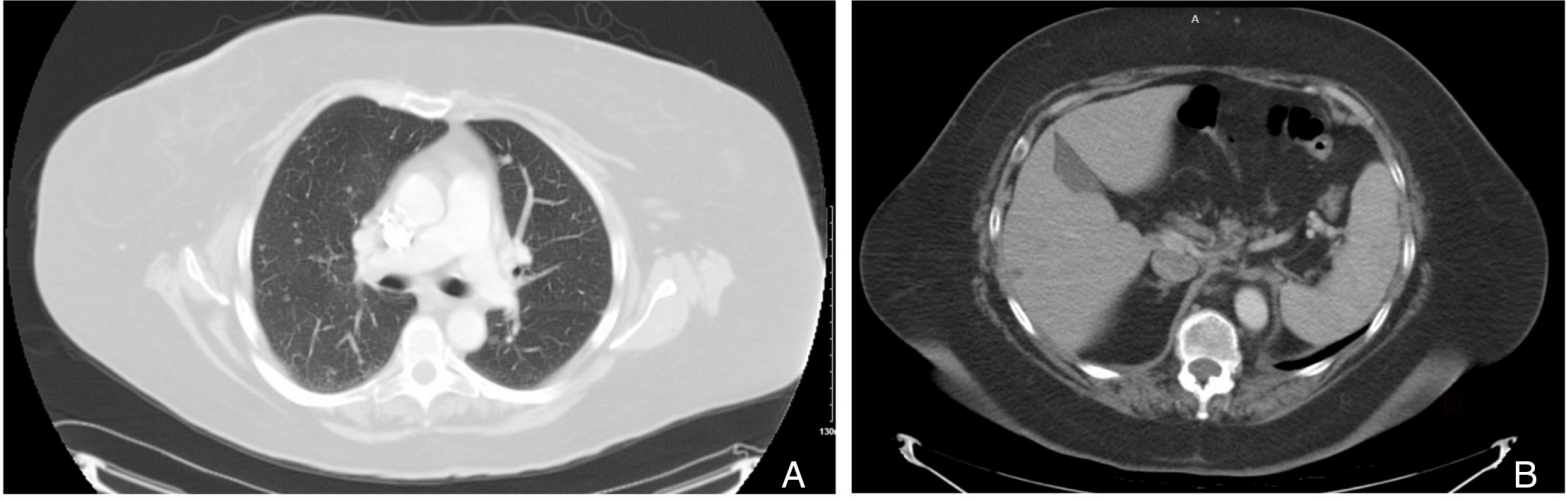
(A) Regression of pulmonary nodules with near‐resolution of mediastinal adenopathy after three cycles of chemotherapy. (B) Near‐complete resolution of the hepatic lesion after chemotherapy.

She began carboplatin–paclitaxel every 3 weeks. After three cycles, CT showed marked regression of pulmonary nodules, near‐resolution of mediastinal adenopathy (Figure 2A), and shrinkage of hepatic lesions (Figure 2B), with resolution of dyspnea. After six cycles, restaging confirmed a very good partial response. She was transitioned to maintenance anastrozole, with subsequent surveillance scans showing stable residual nodules and sustained clinical benefit.

## 3 Discussion

This case underscores an unusual presentation of endometrial carcinoma, with cannonball pulmonary metastases as the first clinical manifestation. Endometrial carcinoma is typically confined to the uterus at diagnosis, and postmenopausal bleeding remains the most common presenting symptom. Typical metastatic sites include local pelvic recurrence, pelvic and para‐aortic nodes, peritoneum, and lungs. Only 15%–20% of patients present with extrauterine spread, and fewer than 5% present with distant metastases at initial diagnosis [3]. Pulmonary involvement, when it occurs, is usually seen in recurrent disease rather than at initial presentation. Thus, widespread lung metastases in the form of large, discrete nodules represent an exceptional diagnostic scenario.

“Cannonball lesions” are radiographically defined as multiple, bilateral, well‐circumscribed nodules, often peripheral, ranging from millimetres to several centimetres in size. They generally indicate hematogenous dissemination and are most often linked to renal cell carcinoma or choriocarcinoma [5, 6]. Although infectious or inflammatory disorders such as tuberculosis, fungal infections, echinococcosis, or granulomatosis with polyangiitis may mimic this appearance, the clinical context and ancillary findings usually help differentiate these from metastatic malignancy [7, 8]. In endometrial carcinoma, venous invasion allows tumour emboli to reach the pulmonary vasculature [3]. The reasons some patients develop well‐circumscribed cannonball nodules rather than diffuse micronodular disease remain unclear, but tumour biology, angiogenesis, and host immune factors are likely contributors.

Molecular characterisation provides important therapeutic guidance. Our patient's tumour was ER/PR positive and MMR‐proficient. Hormonal responsiveness supports the use of agents such as anastrozole, while MMR proficiency excludes eligibility for checkpoint inhibitor immunotherapy currently approved in MMR‐deficient disease [9]. Additionally, PTEN and PIK3R1 alterations implicate PI3K/AKT/mTOR signalling, a pathway under investigation for targeted therapies [10].

The backbone of treatment for stage IV endometrial carcinoma remains systemic chemotherapy, most commonly carboplatin and paclitaxel [11]. Hormonal therapy may be considered in ER/PR‐positive disease either as maintenance or when chemotherapy is poorly tolerated [11]. Surgery has limited value in disseminated disease but can provide symptom control in selected patients. Emerging strategies include immunotherapy and targeted agents tailored to molecular subgroups, offering hope for improved outcomes in the future.

In this case, the patient achieved meaningful clinical and radiographic improvement with chemotherapy followed by hormonal maintenance, consistent with literature showing that systemic therapy can provide both palliation and durable disease control even in advanced presentations.

## Author Contributions

All the authors contributed to the manuscript. Maneesh Gaddam and Midila Bapineni drafted the initial manuscript. All authors listed critically reviewed, edited the manuscript, and approved the version to be published.

## Consent

The authors declare that written informed consent was obtained for the publication of this manuscript and accompanying images and attest that the form used to obtain consent from the patient complies with the Journal requirements as outlined in the author guidelines.

## Conflicts of Interest

The authors declare no conflicts of interest.

## Data Availability

Data sharing not applicable to this article as no datasets were generated or analysed during the current study.
